# Interstitial Lung Diseases and Non-Small Cell Lung Cancer: Particularities in Pathogenesis and Expression of Driver Mutations

**DOI:** 10.3390/genes15070934

**Published:** 2024-07-17

**Authors:** Fotios Sampsonas, Pinelopi Bosgana, Vasiliki Bravou, Argyrios Tzouvelekis, Foteinos-Ioannis Dimitrakopoulos, Eleni Kokkotou

**Affiliations:** 1 Department of Respiratory Medicine, Medical School, University of Patras, 26504 Patras, Greece; atzouvelekis@upatras.gr; 2 Department of Pathology, Medical School, University of Patras, 26504 Patras, Greece; bosgana.p@gmail.com; 3 Department of Anatomy, Embryology and Histology, Medical School, University of Patras, 26504 Patras, Greece; vibra@upatras.gr; 4 Department of Oncology, Medical School, University of Patras, 26504 Patras, Greece; fodimitrak@yahoo.gr; 5 Oncology Unit, The Third Department of Medicine, Medical School, National and Kapodistrian University of Athens, 15772 Athens, Greece; elenkokk@yahoo.gr

**Keywords:** NSCLC, driver mutations, IPF

## Abstract

Introduction: Interstitial lung diseases are a varied group of diseases associated with chronic inflammation and fibrosis. With the emerging and current treatment options, survival rates have vastly improved. Having in mind that the most common type is idiopathic pulmonary fibrosis and that a significant proportion of these patients will develop lung cancer as the disease progresses, prompt diagnosis and personalized treatment of these patients are fundamental. Scope and methods: The scope of this review is to identify and characterize molecular and pathogenetic pathways that can interconnect Interstitial Lung Diseases and lung cancer, especially driver mutations in patients with NSCLC, and to highlight new and emerging treatment options in that view. Results: Common pathogenetic pathways have been identified in sites of chronic inflammation in patients with interstitial lung diseases and lung cancer. Of note, the expression of driver mutations in EGFR, BRAF, and KRAS G12C in patients with NSCLC with concurrent interstitial lung disease is vastly different compared to those patients with NSCLC without Interstitial Lung Disease. Conclusions: NSCLC in patients with Interstitial Lung Disease is a challenging diagnostic and clinical entity, and a personalized medicine approach is fundamental to improving survival and quality of life. Newer anti-fibrotic medications have improved survival in IPF/ILD patients; thus, the incidence of lung cancer is going to vastly increase in the next 5–10 years.

## 1. Introduction

Interstitial lung diseases (ILD) constitute a heterogenous group of chronic lung diseases, characterized by inflammation and fibrosis [1]. ILDs are disorders of lung parenchyma that lead to respiratory failure and end-stage lung disease, with significant morbidity and mortality [2,3]. Computerized Tomography (CT) and histological findings are of cornerstone importance for the diagnosis of ILDs, with high-resolution computed tomography (HRCT) being the most readily available and specific tool [4]. The histological identification of the type of interstitial lung disease is made with a lung biopsy. Transbronchial lung cryobiopsy is proposed as an alternative to surgical biopsy, with comparable diagnostic results but significantly less morbidity and post-procedural mortality and costs [5]. With emerging and current treatment options, survival rates have improved. Newer anti-fibrotic medications have improved survival in IPF/ILD patients; thus, the incidence of lung cancer is going to vastly increase in the next 5–10 years.

## 2. Classification of Interstitial Lung Disease

The last ATS/ERS consensus classification of idiopathic interstitial pneumonias is based on the 2013 Travis et al. classification, with some new concepts by Raghu et al. being updated in 2022 [6,7] (Table 1). The three major groups are: major, rare, and unclassifiable idiopathic interstitial pneumonias. The major idiopathic interstitial pneumonias include Idiopathic Pulmonary Fibrosis (IPF), Idiopathic non-specific Interstitial pneumonia (NSIP), Respiratory bronchiolitis-interstitial lung disease (BR-ILD), Desquamative Interstitial pneumonia (DIP), Cryptogenic organization pneumonia (COP) and Acute interstitial pneumonia (AIP). The rare idiopathic interstitial pneumonias contain two types: Idiopathic lymphoid interstitial pneumonia (LIP) and Idiopathic pleuro-parenchymatous fibroelastosis. At last, unclassifiable idiopathic interstitial pneumonia is a categorization that includes cases that have inadequate clinical, radiological, or pathological information (previous therapy, different patterns in CT, etc.) [6].

The new concepts consist of the following terms: Interstitial pneumonia with autoimmune features (IPAF) [8,9], Progressive fibrosing interstitial lung diseases (PF-ILD) [10,11] and Interstitial lung abnormalities (ILAs) [12], and they define patients that do not fulfill the criteria of the above terms. 

## 3. Idiopathic Pulmonary Fibrosis

Idiopathic pulmonary fibrosis (IPF) is the first or second most common type of ILD [13,14]. It is more frequent in males in the sixth or seventh decades of life, and the clinical image is increasing dyspnea and distortion of lung function, with a poor prognosis [15]. It is characterized by the histological pattern of usual intestinal pneumonia [13]. The major pathologic findings are patchy dense fibrosis with architectural distortion, predilection for subpleural and paraseptal lung parenchyma, fibroblastic foci, and the absence of features that suggest an alternate diagnosis [4,13]. The cause of idiopathic pulmonary fibrosis is still unknown.

**Figure 1:**
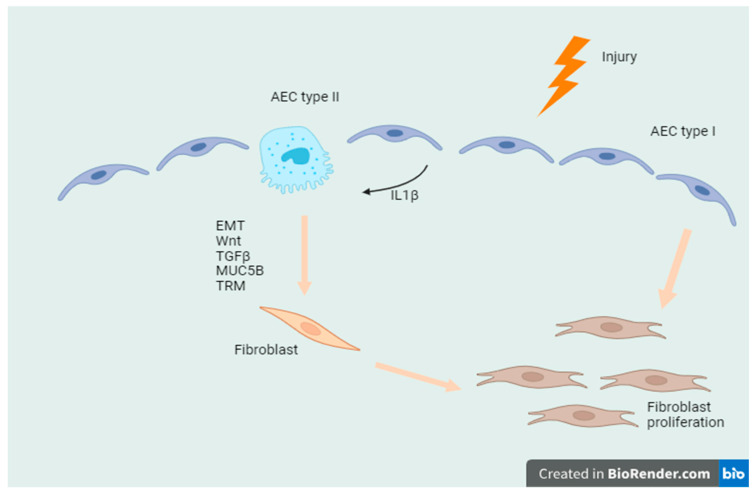
Pathogenetic mechanism of IPF. Injury and molecular events lead to abnormal fibroblast proliferation and pulmonary fibrosis.

The pathogenesis of IPF is a complex process that includes responses to epithelial damage and several molecular mechanisms that lead to fibrosis [16]. The recurrent alveolar epithelial cell (AEC) injury model is the main pathogenetic mechanism that is described. Predisposing factors, such as genetic, environmental, epigenetic, and immunologic, cause epithelial damage and dysregulated epithelial repair [17]. This results in abnormal fibroblast proliferation, dilatation of the extracellular matrix, and loss of normal lung architecture [16] (Figure 1).

The molecular events that lead to IPF orchestrate all cell types that participate in epithelial injury repair and lung fibrosis (alveolar epithelial cells, fibroblasts, endothelial cells, and immune cells) [17].

Data from scRNA-seq comparing IPF and control lung tissue have provided evidence for the presence of an aberrant epithelial cell type, which expresses markers including matrix metalloproteinase 7 (MMP7), integrin αVβ6, cellular senescence, and epithelial–mesenchymal transition (EMT) and represents a major driver of IPF pathogenesis [18].

IL-1β is important in the normal differentiation of AT2 cells into AT1 cells. IL-1β signaling is another mechanism by which chronic inflammation leads to fibrosis [19].

### 3.1. Wnt/β Catenin Signaling Pathway

The Wnt signaling pathway and its nuclear mediator β-catenin are involved in the development of normal lung tissue and organogenesis [20]. It plays a crucial role in the differentiation of the normal bronchial and alveolar epithelial cells [21]. Alveolar type 2 progenitor cells (AT2) control normal lung degeneration and repair in response to lung injury [22]. The Wnt pathway is implicated in the pathogenesis of pulmonary fibrosis [23]. After injury, fibroblasts maintain AT2 cells through Wnt signaling. Sustained Wnt signaling through β-catenin inhibits AT2 cell differentiation and leads to lung fibrosis [24,25]. AT2 cells prevail in the expression of IL-1β and cause fibrosis through TGF-β [26]. It is also described the role of β-catenin signaling in the epithelial–mesenchymal transition (EMT), which is important in embryonic development, tumor progression, and fibrosis [27,28].

Epithelial–mesenchymal transition (EMT) is a step in normal injury repair and is characterized by the loss of cell-to-cell adhesion molecules, such as E-cadherin and is regulated in IPF and cancer [29,30]. EMT is probably a process where epithelial cells differentiate into mesenchymal cells and promote local fibrosis [31].

### 3.2. Notch Signaling Pathway

The Notch signaling pathway is important in the normal development of alveolar epithelium and controls SOX2 signaling, which promotes the honeycomb appearance of alveolar epithelium [32,33]. Dysregulation of this pathway is observed in alveolar epithelial cells in IPF, and it is evident that it ruins alveolar epithelial differentiation and promotes fibrosis [34].

### 3.3. TGF-β Signaling Pathway

**Figure 2:**
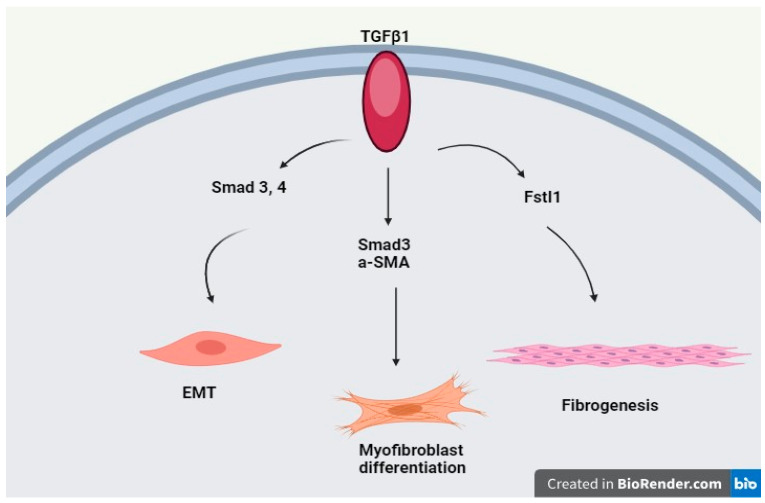
The role of the TGFβ1/Smad signaling pathway in idiopathic pulmonary fibrosis.

Transforming growth factor-β (TGF-β) is the factor that has a role in fibrosis. In normal healing after injury, fibroblasts, differentiation of myofibroblasts, and deposition of extracellular matrix take place in order to close the trauma [35]. Upregulation of the TGF-β signaling pathway is observed in fibrotic diseases, which promotes activation of fibrotic mesenchymal cells and deposition of extracellular matrix. TGF-β signaling has been involved in pulmonary fibrosis [36,37]. TGF-β1 activates HMGB1, RELM-β, Slit2, and Fstl1 by cooperating with Smad2 and Smad3 and influences the three steps of idiopathic pulmonary fibrosis (EMT, myofibroblast differentiation, and fibrogenesis) [38] (Figure 2).

### 3.4. MUC5B

Familiar pulmonary fibroses represent about 5–20% of cases with IPF [39]. This fact suggests that molecular events are implicated in the pathogenesis of the disease. The most common mutation is the MUC5B risk allele [40]. The MUC5B promoter polymorphism is related to mucociliary clearance epithelial activity and is associated with interstitial lung disease [41,42]. Overexpression of MUC5B leads to mucus hypersecretion in bronchoalveolar epithelium and promotes inflammation and injury [43].

### 3.5. TOLLIP

Toll-interacting protein is an intracellular adaptor protein that has a role in inflammation, immune response, and lung epithelial cell apoptosis [44]. Polymorphism in the promoter for the Toll-interacting protein (TOLLIP) is another genetic event that is associated with an increased risk of developing IPF [45]. These genetic variants provide novel advantages for targeted therapies in IPF [17,43].

### 3.6. Telomerase-Related Mutations (TRM)

Telomeres are nucleotide sequences that stabilize chromosome edges in order to prevent chromosome shortening during cell replication [46]. Telomerase-related mutations (TRM) are observed in familiar and sporadic IPF [47]. Several mutations of this protein complex have been described, with TERC and TERT being the most common [47,48].

## 4. ILD and NSCLC

Patients with ILD have an increased risk of developing lung cancer [49,50,51,52,53]. In a metanalysis of 35 studies, 13.5% of patients with IPF developed non-small cell lung cancer (NSCLC). Squamous cell carcinoma was the most frequent histologic variant (37.8%), in contrast to adenocarcinoma (30.8%) [50]. A study of 103 patients with IPF showed that the age of diagnosis of the disease was associated with the risk of lung cancer [54]. In a study of 938 patients with IPF, males and smokers had an increased risk for lung cancer development [55]. ILD and systemic sclerosis are associated with lung carcinogenesis [51].

The presence of lung cancer in patients with IPF is associated with worse survival [56]. Furthermore, patients with ILD have greater lung cancer-specific mortality and shorter overall survival in stage I non-small cell lung cancer (NSCLC) [57]. Combined pulmonary fibrosis and emphysema are associated with an increased risk of developing squamous cell carcinoma, especially in the case of male smokers, with a median survival rate of 19.5 months [58].

There are several common molecular events that are observed in lung cancer and in patients with ILD. Mutations in the TP53 gene have been found in patients with squamous cell carcinoma of the lung and IPF [59,60,61]. Decreased PTEN expression and hyperactivation of Akt have been discovered in the alveolar epithelial cells and fibroblastic areas of human IPF lungs and patients with lung cancer [62]. That makes PTEN/P13K/Akt signaling a crucial pathogenetic mechanism and a potential therapeutic target for IPF and NSCLC [63]. Telomerase reverse transcriptase (TERT) and telomerase RNA (TERC) are activated in lung cancer [64,65]. Mutations of the TERT and TERC genes are associated with IPF as well [66,67].

Cancer-associated fibroblasts (CAFs) are specific cell types that determine tumors biological behavior. These are hot spots in immunotherapy for tumors and targeted therapy [68]. These results suggest the need for more research to discover the mechanisms linking pulmonary fibrosis and lung carcinogenesis. The treatment of patients with IPF and lung cancer is challenging because surgery, radiation therapy, or chemotherapy may cause exacerbations of the underlying ILD, which can lead to complications and death [51,58]. Thus, it is important to find targeted therapies with fewer complications for these patients in order to decrease morbidity and mortality.

## 5. Driver Mutations in Patients with NSCLC and ILD

Lung Cancer (LC), as already mentioned, is a very common comorbidity in patients with ILDs, especially IPF, with reported cumulative incidence rates reaching 15.9% and 31.1% at 5 and 10 years, respectively, which is significantly higher than in the general population [55]. Additionally, in patients with IPF and LC, the mean survival time seems to be significantly shorter (1.6–1.7 years) than in those who had no LC. This is also the case for patients with non-IPF ILDs like cryptogenic organizing pneumonias [69,70].

Recent studies have highlighted that in terms of physical functionality, ILD clinical characteristics, and physiological parameters, no major differences are encountered in patients with lung cancer and ILDs versus those with ILDs alone [69]. Nevertheless, higher rates of Forced Vital Capacity decline (more than 10% annually), male gender, increased age, combined pulmonary emphysema and fibrosis, and active smoking were clinical and epidemiological parameters independently associated with increased LC incidence rates [58,71].

In patients with IPF and other ILDs, the incidence of Small Cell Lung Cancer seems to be relatively lower than in the general population, with NSCLC (AdNSCLC and sqNSCLC) being the most common subtypes, with the latter exhibiting slightly increased incidence rates in some studies [55], but not in others [72]. Areas of dense fibrosis/bronchiolectasis, and honeycombing are the predominant sites of lung cancer development in those patients. Lower lobes, rather than the upper lobes, are also more frequently affected, in at least 50% of cases [72,73].

The latency time from the diagnosis of IPF to the final diagnosis of LC varies, but in the vast majority of the cases, it ranges from 12 to 36 months. HRCT is the diagnostic modality of choice in order to diagnose suspicious lesions in IPF patients, since it is also routinely, annually, or biannually used to monitor patients with ILDs. No differences are noticed in the diagnostic pathway in patients with LC and IPF vs. those without IPF [72].

Real-life data show that available treatment modalities do not differ between patients with LC/IPF and LC alone, but the applicability of these greatly depends on patients’ clinical performance, ILD characteristics, and respiratory physiological reserves [72]. Surgery remains a valid option for patients with early-stage disease, but special precautions in terms of the anesthesia protocols that can be applied should be taken. It is well established that acute exacerbations of the underlying ILD might occur following the surgery [74]. Patients that are not candidates for surgical treatment and harbor actionable driver mutations like EGFR, ALK, BRAF, etc. are good candidates for targeted treatment. Nevertheless, these treatments are also related to acute ILD exacerbations, with incidence ranging from 4% for gefitinib to 2.14% with anti-ALK targeted treatments, especially in Japanese populations, whereas moderate and severe Grade 5 pneumonitis is significantly less prevalent in non-Asian populations [75,76,77,78]. Therefore, targeted therapies remain a viable option for treatment in patients with ILDs, with special considerations for shorter follow-up to be applied in Asian populations.

As already mentioned, the pathogenetic mechanism of interstitial lung disease and, in particular, idiopathic pulmonary fibrosis includes different pathological and molecular peculiarities and has a great heterogeneity [79]. Nevertheless, some IPF features are implicated in cancer [80]. Proteins related to inflammation, such as TGFβ-1, confer a risk for IPF and are associated with cancer as well [81,82]. Recent studies showed that driver mutations are implicated in cases of non-small cell lung cancer and interstitial lung disease. Patients with IPF have a high risk of developing squamous cell lung cancer (sqNSCLC) [83]. A recent exon sequencing study of 47 human lung cases with sqNSCLC, with or without IPF, has revealed the genetic alterations in patients with sqNSCLC and IPF. The common mutations that are observed in patients with sqNSCLC and IPF are SETD2 and NFE2L2 mutations and MYC amplification [84]. Particularly, the presence of the SETD2 mutation in patients with sqNSCLC and IPF is associated with a poorer prognosis [84]. SETD2 belongs to the nuclear receptor SET domain family of methyltransferases, which methylates the lysine 36 of demethylated histone H3 (H3K36me2) [85]. Dysregulation of SETD2 methyltransferase, by mutation or loss of function, is noticed in many solid tumors and in non-small cell lung cancer [85]. Nuclear factor erythroid-derived 2, like 2 (NFE2L2) is a transcription factor that plays a role in antioxidant enzyme regulation [86]. Mutations of this factor are observed in patients with sqNSCLC and IPF as well [83]. Particularly, knockdown of the NFE2L2 gene is observed in lung cancer with a prognostic value [86]. Loss of NFE2L2 leads to oxidative stress, which plays a role in lung carcinogenesis. Induction of NFE2L2 might be a therapeutic target for NSCLC and IPF [86]. MYC amplification is associated with cases of NSCLC and IPF [83,87].

Normal alveolar epithelial cells can transform into fibroblasts, with an epithelial-to-mesenchymal transition, in response to TGFβ signaling, as already mentioned. This transformation is a mechanism that leads to idiopathic pulmonary fibrosis [88]. It has been reported that the EMT of EGFR-mutant NSCLC is associated with TKI resistance [89]. There is strong evidence that EMT of non-neoplastic alveolar epithelial cells plays a role in fibrosis in idiopathic pulmonary fibrosis [90].

The epidermal growth factor receptor (EGFR) is a member of the family of ErbB tyrosine kinase receptors, which is also known as ErbB1 or HER1 [91]. This receptor is involved in many normal developmental and physiological processes [92]. ErbB signaling is involved in many types of malignancies, including lung, breast, glioblastomas, and ovarian cancer [93,94,95]. The EGFR signaling pathway is involved in lung fibroses in some recent studies in mice, who express TGF-a in alveolar epithelial cells and show progressive fibrosis [96]. This gives hope that EGFR-targeted therapy may have good results in patient’s lung fibrosis [97]. Also, the HER1 ligand amphiregulin and the EGFR signaling pathway are observed in cases with TGFb1-dependent pulmonary fibrosis [98]. Surprisingly, KRAS mutations, including the G12C variant that can be treated with sotorasib and adagrasib, were identified in the metaplastic bronchiolar epithelium in patients with honeycomb (UIP) lesions, highlighting the pathological continuum from metaplasia to lung cancer in this group of patients [99].

A recent study assessed the immunohistochemical expression of EGFR, showing positive pEGFR expression in the myofibroblastic foci of lung samples. These results indicate that an EGFR mutation burden is observed in lung fibroblasts in patients with IPF [100]. It is already known that smoking is a negative predictive factor for patients with EGFR mutations. The presence of ILD is a negative indicator for carrying EGFR mutations in patients with lung adenocarcinoma [101]. A study by Guyard and colleagues has also shown that there is an opposite association between patients with ILD and tumors with EGFR mutations. Only one patient (1.8%) with aNSCLC was harboring an EGFR mutation, while the majority had TP53 (64.5%), BRAF (9.7%), MET (12.9%), and KRAS (3.2%) mutations, while no ALK or ROS1 mutations were identified [100]. These low rates of EGFR mutations in patients with ILD were replicated in a study by Honda et al., where the prevalence of EGFR, KRAS, and BRAF driver gene mutations was 1.9, 20.4%, and 3.7%, respectively [102]. In an older study with highly selected patients with smoking-related IPF, the prevalence of driver mutations in EGFR and KRAS was 14.1% and 37.5%, respectively, showing consistency for the high KRAS mutation rates with the studies of [103,104], and the aforementioned studies by Honda et al. and Fujimoto et al. [105,106]. Of note in the study by Hwang et al., BRAF mutations accounted for 17.1% of cases (6/35), which is higher than the rates reported in the literature [107,108].

## 6. Conclusions

Numerous studies have shown that multiplex gene testing is usually not performed in patients with severe comorbidities, like patients with concurrent ILDs, even though these patients may harbor KRAS, G12C, and BRAF mutations that could subsequently be treated by current and emerging targeting therapies, offering survival and quality of life benefits [91,97]. A reason for the lower driver mutation screening rates in patients with ILD and lung cancer could be that the preexisting ILD may be associated with the development of severe drug-induced pneumonitis in NSCLC patients [104,109] treated with tyrosine kinase regimens, but this is not the case for the KRAS G12C.

Having in mind that KRAS G12C and BRAF mutations are expressed in a significant number of ILD/IPF cases with concurrent NSCLC, we must offer thorough genetic testing in these patients beyond EGFR and ALK testing. KRAS G12C mutations have been identified in metaplastic epithelium in honeycomb foci, highlighting the plausible common pathogenetic pathway of IPF and lung cancer. Adagrasib and sotorasib are available for the treatment of KRAS G12C mutations, and they have acceptable pneumonitis rates, which can therefore be a game changer in these patients. Nevertheless, more studies are needed in that view to determine the underlying pathophysiological mechanisms of the expression of driver mutations in patients with concurrent ILDs and NSCLC and identify those that can be offered targeted treatment.

## Figures and Tables

**Table 1:** Classification of idiopathic interstitial pneumonias (Updated 2022/6-7]).

Major	Idiopathic Pulmonary Fibrosis (IPF)
	Idiopathic non-specific Interstitial pneumonia (NSIP)
	Respiratory bronchiolitis-interstitial lung disease (BR-ILD)
	Desquamative Interstitial pneumonia (DIP)
	Cryptogenic organization pneumonia (COP)
	Acute interstitial pneumonia (AIP)
Rare	Idiopathic lymphoid interstitial pneumonia (LIP)
	Idiopathic pleuro-parenchymatous fibroelastosis
Unclassifiable	
New concepts	Interstitial pneumonia with autoimmune features (IPAF)
	Progressive fibrosing interstitial lung diseases (PF-ILD)
	Interstitial lung abnormalities (ILAs)

## Data Availability

Not applicable.
